# ‘I Didn’t Know Where to Go’: A Mixed-Methods Approach to Explore Migrants’ Perspectives of Access and Use of Health Services during the COVID-19 Pandemic

**DOI:** 10.3390/ijerph192013201

**Published:** 2022-10-13

**Authors:** Ana Gama, Maria J. Marques, João Victor Rocha, Sofia Azeredo-Lopes, Walaa Kinaan, Ana Sá Machado, Sónia Dias

**Affiliations:** 1 NOVA National School of Public Health, Public Health Research Centre, Universidade NOVA de Lisboa, 1600-560 Lisboa, Portugal; 2 Comprehensive Health Research Centre (CHRC), Universidade NOVA de Lisboa, 1169-056 Lisboa, Portugal; 3 Statistics and Operational Research Department, Sciences Faculty, University of Lisbon, 1749-016 Lisboa, Portugal

**Keywords:** migrants, health services utilization, COVID-19 pandemic, healthcare inequalities

## Abstract

The COVID-19 pandemic put pressure on health systems, affecting populations’ use of health services, especially those experiencing increased difficulties in healthcare access, as some migrant groups. This study aimed to investigate access and use of health services during the COVID-19 pandemic among migrants in Portugal. A mixed-methods approach was used. A community-based cross-sectional survey was conducted involving migrant communities residing in the Lisbon Metropolitan Area. Analyses of a subsample of participants (*n* = 929) examined factors associated with perceived worsening of access to health services during the pandemic. Semi-structured interviews with 14 migrants were conducted and thematically analyzed to further understand experiences and difficulties in health services’ use. Around 44% of surveyed participants reported worsening of access to health services since the pandemic, more frequently women, those with lower income, and those who perceived being at moderate or high risk for COVID-19 infection. Digital change in services and lack of formal and informal support during lockdowns were highlighted by interviewers as main barriers in access to healthcare for migrants. The pandemic renewed concerns about inequalities in healthcare access among migrants. It is key that in following years health systems are able to address the potential accumulated burden of disease.

## 1. Introduction

The emergence of SARS-CoV-2 and the consequent COVID-19 pandemic put severe pressure on health systems worldwide, with significant changes in demand for inpatient care [1]. Governments all over the world were forced to implement measures to increase the national health services’ capacity to respond to the outbreak, besides staying at home, social distancing, and lockdown measures. In many countries, several outpatient and inpatient care services were suspended to reallocate health professionals and other resources to COVID-19 care. In several hospitals worldwide, such as in Portugal, all non-urgent elective surgeries were canceled to increase bed capacity and free up ventilators for COVID-19 patients [2,3,4,5]. Face-to-face appointments decreased or were replaced by telephone/online format to minimize the risk of disease transmission and the threat to health professionals [6,7]. In addition, research has shown that many patients avoided seeking health care for fear of contamination by COVID-19 [4,5,7].

This disruption in services left many patients without access to health care. There were reports of a significant reduction in the use of emergency services throughout the pandemic when compared to similar periods of previous years [3,4,8,9], as well as a substantial decrease in primary care consultations [10,11,12,13]. Furthermore, evidence shows that the pandemic has affected populations asymmetrically, potentially accentuating inequalities [14]. Particularly vulnerable populations include those in more adverse socioeconomic conditions, at greater risk of income loss and unemployment, and with increased difficulties in accessing health services [15,16], as are some migrants.

The literature shows that most migrant populations tend to have a good health status on arrival in the host country compared to the native populations, but this advantage can diminish over time as exposure to health risks increases [17,18]. Upon arrival in host countries, some groups of migrants face a new physical and social environment that, combined with fragile socioeconomic conditions, contributes to an increased vulnerability in health [18]. In addition, studies indicate that some groups tend to experience difficulties in accessing health services, especially those more socially vulnerable, such as recently arrived migrants and those without a regular migration status [18,19,20,21,22,23,24]. Cultural and linguistic differences, economic difficulties, lack of social support, stigma, and discrimination can also hamper access to available information and health care.

The existing literature suggests that migrant populations’ access to health care may have been further affected in the context of the COVID-19 pandemic and the restrictions of health services [25,26]. Indeed, a recent study conducted in the UK showed a decrease in appointments among most vulnerable migrants during the pandemic, which resulted from the shift of health services into remote format [27]. According to some authors, the pandemic increased existing inequalities in healthcare access for vulnerable migrants, with those in the greatest need of healthcare having less access to digital technologies and information [27,28]. Despite growing research on the impact of the pandemic on migrant populations, the specific impact of the pandemic on the access and use of health services among migrants had not yet been explored in depth, and less is known about migrants residing in Portugal. Knowledge about the migrants’ experiences in access and barriers to healthcare during the pandemic from their point of view is crucial to inform tailored strategies that support them in accessing health services. Simultaneously, it can help improve the provision of healthcare and promote health equity. This paper contributes with new knowledge about migrants’ experienced needs in access to healthcare. Access to health-care has been defined by numerous conceptual frameworks as a multidimensional concept, generally involving health systems and patients-related multilevel factors [29,30,31,32,33]. This study explores access to healthcare in the context of health services’ availability and populations’ ability to seek and reach health care, as defined in Levesque’s conceptual framework for access to health care [33]. Based on quantitative and qualitative data collected among migrants in Portugal, the aim of this study was to investigate access and use of health services during the COVID-19 pandemic among these populations.

## 2. Materials and Methods

This study used a mixed-methods approach, and employed a sequential explanatory design [34], which consisted of a quantitative component (survey), followed by a qualitative component (semi-structured interviews) conducted with migrants. This research was approved by an ethical committee.

### 2.1. Survey

A cross-sectional survey was conducted in the early phases of the pandemic among migrants residing in Portugal [35]. The survey was carried out in the Lisbon Metropolitan Area, an area with a high density of migrants. In 2020, Portugal hosted 662,095 foreign citizens [36], of whom 43% resided in Lisbon. The study included migrants aged 18 years or older, who were born in a Portuguese-speaking African, Middle Eastern, or Asian country, or Brazil, who had lived in Portugal for less than 10 years and who were currently residing in the Lisbon Metropolitan Area.

As there was not a sampling frame that could be used to draw a representative sample of migrants in the Lisbon Metropolitan Area that included most socially vulnerable groups (e.g., recently arrived, undocumented), a community-based sampling approach was used. Migrants were recruited and invited to participate in the study through key stakeholders (e.g., nongovernmental and governmental organizations, migrant associations) regardless of their health condition and migration status. Informal migrant community leaders were also invited to collaborate as recruiters of potential participants within their social networks. Data were collected through a ‘pen-and-paper’ questionnaire administered by trained bilingual researchers. The questionnaire was pre-tested and was made available in Portuguese, English, Arabic, Bengali, Hindi, Mandarin, and Nepali, with an estimation of 10 min to be completed. The questionnaire included items on sociodemographic characteristics, such as age (measured by date of birth), sex, educational level, monthly household income, and living arrangements. Data were also collected on post-migration-related characteristics, such as length of stay (‘<1 year’, ‘1 to 5 years’, ‘6 to 10 years’, ‘over 10 years’), Portuguese language proficiency and migration status, and on self-perceived risk to get COVID-19 infection (‘high risk’, ‘moderate risk’, ‘low risk’, ‘no risk’, ‘do not know’, ‘prefer not to answer’). Perceived change in access to health services during the pandemic was measured by the question “Since the coronavirus crisis, how is your access to health services?”, with three response options: “Got worse”, “The same as before” and “Got better”. In total, 1126 migrants participated in the survey. For the present study, analyses focused on the subsample of participants with valid responses to the question on perceived change in access to health services (*n* = 929).

#### Quantitative Data Analysis

Descriptive analysis of the sample characteristics was performed and stratified by perceived change in access to health services during the pandemic. The factors associated with this dependent variable were examined through logistic regression models; data were presented as crude odds ratios (ORs), adjusted odds ratios (aORs), and 95% Confidence intervals (95% CI). Statistical significance was set at a value of *p* < 0.05. Data analysis was performed using the R software version 4.0.2 (R Foundation for Statistical Computing, Vienna, Austria, 22 June 2020).

### 2.2. Semi-Structured Interviews

Semi-structured individual interviews were conducted with 14 migrants who met the inclusion criteria mentioned previously. Participants were recruited through third-sector organizations that provide services for migrant communities in the Lisbon Metropolitan Area. The interviews were conducted in a private space, in Portuguese or English, according to the participant’s preference. The interview guide covered the topics of migrants’ perceived health care needs, and facilitators to accessing health services during the COVID-19 crisis.

#### Qualitative Data Analysis

A thematic analysis approach was used. Interviews were audio recorded and then transcribed and analyzed thematically. One researcher conducted the initial qualitative analysis using the Braun and Clarke’s six-phase thematic analysis process [37]: ata familiarization, generating initial codes, search for themes and sub-themes, reviewing themes, defining and naming themes, and writing the findings. Following a deductive approach, the initial codes and themes for the qualitative data analysis were defined based on a preliminary literature review and the Levesque’s conceptual framework for access to healthcare [33] which underlies the study, as well as the descriptive analysis of the survey data. The themes of analysis comprised perceived impact of the pandemic in health, quality of life and access to health care, experiences in seeking health care, perceived facilitating factors and barriers in access to health services. These themes further evolved into subthemes during the analysis of the interviews. Data were converted into segments of relevant information and concepts, then organized into the themes and subthemes, and the results were analyzed and interpreted. The internal homogeneity of the themes was largely verified by the aforementioned researcher. Whenever questions emerged during the analysis, e.g., regarding the coding scheme and the ‘best fit’ of some excerpts, a discussion was held with a second researcher until a consensus was reached. The external homogeneity was assessed by the two researchers. Quotes were chosen to illustrate the topics, meanings, and contexts provided by the interviewees.

## 3. Results

### 3.1. Survey Results

#### 3.1.1. Characteristics of the Participants

Table 1 presents the characteristics of the surveyed participants. The proportion of men and women was 45.5% and 54.5%, respectively. Most of the participants were between 26–45 years old (66.5%), 42.7% of them had secondary education, and 65.2% had a monthly household income below 650€ (national minimum wage). About 43% were living with their nuclear family, 25.6% with other people, and 20.3% were living alone. Overall, 83.6% of the participants had lived in Portugal for <5 years and 18.4% had arrived during the last year; over two-thirds reported being able to understand and speak the Portuguese language. Around 91% were documented or in the process of regularization and 8.7% were undocumented. When asked about their perceived risk to get COVID-19, one participant in five considered it to be high. Around 48% were born in a Portuguese-speaking African country, 26.8% in a Middle Eastern or South Asian country, and 21.6% in Brazil.

#### 3.1.2. Perceived Change in the Access to Health Services since the COVID-19 Pandemic

Participants were asked about their access to health services since the COVID-19 pandemic began. Overall, 56.3% reported that the access remained the same or better than before, but for 43.7%, the access to health services got worst (Table 2). The multivariable analysis showed that the prevalence and odds of perceiving a worsening of the access to health services were higher among women (aOR = 1.41, CI 95% 1.02–1.97), those who perceived moderate or high risk for COVID-19 (aOR = 1.64, CI 95% 1.12–2.41 and aOR = 1.59, CI 95% 1.02–2.46, compared to no/low risk), and those who had a monthly household income lower than 650€ (aOR = 1.43, CI 95% 1.01–2.03).

### 3.2. Semi-Structured Interviews Results

#### 3.2.1. Characteristics of the Participants

Out of the 14 interviewed participants, 8 were women, 7 were between 26–45 years old, 5 were older, and 2 were younger. Overall, 4 interviewees had basic education (i.e., nine or fewer years of education), 6 had secondary education, 4 had higher education, and 7 were unemployed. Overall, 12 participants had lived in Portugal for less than five years, 10 were documented or in the process of regularization, and 11 reported being able to understand and speak Portuguese. The participants were from a Portuguese-speaking African country (*n* = 5), an Asian country (*n* = 5) and Brazil (*n* = 4).

#### 3.2.2. Healthcare Seeking during the COVID-19 Pandemic

Some participants reported that during the pandemic, they avoided seeking health services due to fearing the risk of contamination by COVID-19. Lack of formal and informal support after the establishment of the mandatory lockdown measures were also reported reasons for not using the health services for migrants with children:

*“I needed to go to the primary healthcare center because of my diabetes but I had my three small children at home. I’m a single mother. The schools were closed for three months. Because of the lockdown I couldn’t rely on any support from friends or family. What was I supposed to do? Leave them alone?”* (P1)

Some of the interviewees who had moved to Portugal the year before referred to language barriers and the preferential use of hospital emergency services due to a lack of knowledge on how to navigate the health system:

*“I was feeling sick. I didn’t know where to go. I tried to call SNS [National Health Service] without success. I went to the primary care center, but the security guard and the front officer did not speak…or didn’t want to speak in English. Then I asked for help to a neighbor and ended up at the emergency services of the public hospital.”* (P6)

Other interviewees reported that, despite having chronic diseases such as diabetes or hypertension, they needed to secure basic needs first, such as housing or food, due to the loss of income and work. For some of the participants, these additional stressors led to increased feelings of anxiety and depression:

*“When you need to choose between taking care of your health or eating and having a place to sleep at night, what do you go for? I had lost my job during the lockdown. I worked as a housekeeper. My concern was finding food banks or other social supports. I was always worried.”* (P2)

Few migrants reported difficulties in keeping themselves up to date with the frequent changes in health-services and identifying reliable sources of information due to the considerable misinformation about COVID-19:

*“I didn’t know where to go. I thought the emergency services were closed or only served people with COVID.”* (P8)

*“I say there was no information, why? Because every day, or every week, there was new information. So that left people… in my case…a little confused, I never knew what was right and what was wrong.”* (P9)

#### 3.2.3. Perceived Impact of COVID-19 on the Access and Use of Health Services

Interviewees reported both positive and negative impacts of the pandemic on the access and use of health services. On the positive side, most migrants mentioned some measures implemented by the Portuguese Government, such as the grant of residence to immigrants and asylum seekers with pending applications:

*“I arrived some months before the pandemic and immediately received the residence permit after the lockdown. Being documented facilitated access to health services.”* (P10)

Regarding perceived barriers, most of the participants reported a deterioration in the access to healthcare not related to COVID-19, such as routine medical care (e.g., regular check-ups):

*“I needed a prescription for the regular mammogram but couldn’t talk to my doctor. I had breast cancer three years ago. I was really worried.”* (P1)

*“Yes, because everything (health centers) was full, and there was no place, there was only COVID, there was nothing else, it got complicated.”* (P4)

More than half of the interviewees expressed concern about the digital change in services (e.g., in registration, appointments, and provision of health information and prescriptions by SMS) that made it difficult to access healthcare. Some of them shared not knowing how to use these tools, and others lacked resources or privacy at home to use them:

*“Is the internet secure and credible? We are asked to access a website or to download an app, to fill out a form to request a COVID test or a digital certificate, to enter with a digital mobile key… Then we need to print the prescription to get a COVID test. I mean, I don’t have a printer. I can do it online, but most people don’t know how to use the electronic signature. It is easier if you have a computer. Very hard to do it over phone.”* (P12)

*“How can I talk about my health over the phone or on a computer with four more people [coresidents] in a small apartment?”* (P14)

*“I know nothing about computers. My daughter always does that for me […] These things, I don’t know.”* (P9)

Some participants felt that the check-up calls were reassuring, but limited in scope and content:

*“I wish it could be different…When he [GP] calls me…I know the consultations are all now by phone, but he hangs up fast, I don’t have time to share my doubts. I understand that they have a lot of patients but…”* (P3)

Some participants also reported a loss of access to support networks and community organizations during the pandemic, that previously helped them accessing the health care and navigate the health system:

*“Before COVID-19 people from the community and migrants’ associations used to help us, inform us about how to address healthcare costs and access health services. But now everything is more difficult.”* (P5)

Some participants, namely those residing in Portugal for less than a year, reported that lockdowns also reduced the access to informal support (e.g., colleagues, friends, neighbors) who used to help with the language issues. This hampered their ability to access and understand health information, appointment messages and emails about COVID-19:

*“I arrived in Portugal two months before the first lockdown. I don’t speak Portuguese and my English is poor. If I got a message or email from the health services, I couldn’t ask my friends for help in the translation.”* (P10)

## 4. Discussion

The pandemic and the consequent disruptions in health services hindered the access to healthcare in many countries [38], likely with marked disparities among migrant populations. This study allowed insight into the perceived impact of the COVID-19 pandemic on the access and use of health services among migrants in Portugal.

In our study, nearly half of the surveyed participants perceived a worsening in the access to health services during the pandemic. Migrants with lower income had higher chances of perceiving worse access to the health services, suggesting a social gradient in access to care. Despite the national efforts that were taken to extend residence permits and deadlines for the regularization of the migration status in Portugal, as well as the free universal health coverage even for those undocumented, inequalities in healthcare access associated with social conditions still occur.

Our results revealed that the worsening in access to health services was perceived differently between men and women. Research on sex differences in health has shown that women tend to be more concerned about health issues and use more health services than men [35,39,40]. Also, previous research showed that, in comparison with men, migrant women experienced more hurdles in accessing health services during the pandemic, which can be attributed to gendered cultural norms [41] and additional childcare responsibilities, with the closing of schools and less formal and informal support [42].

A self-perception of moderate or higher risk of getting COVID-19 was associated with a perceived worsening in access to health services. Studies in South Africa and the United States of America for the general population also found that a higher-risk perception of getting infected with COVID-19 was associated with a higher probability of avoiding medical care [43,44]. Our survey was conducted at a time when community fear and mistrust were high and there were only a few mitigation strategies to support essential health services continuity [38]. In Portugal, as in other countries, there was also significant uncertainty and misinformation about the pandemic [45], which may have contributed to increase risk perception.

The semi-structured interviews enabled to further explore some of these factors and highlighted barriers to the use of health services. The digital change in healthcare provision, despite being one of the approaches to overcome services disruption in many countries, may have aggravated existing inequalities regarding access, especially for some migrant groups who lacked access to, or capacity to use technology. Concerns about language constraints and establishing a relationship of trust were also mentioned by participants. Indeed, previous research demonstrated that COVID-19 has accelerated remote healthcare provision, with potentially permanent changes that affect disadvantaged populations [46]. A study with migrants in Finland showed that while remote appointments could be effective, they were also considered less personal than face-to-face services and potentially leading to more misunderstandings [47].

In addition, the lack of formal and informal support during lockdowns was described as a hindering factor in access to healthcare. This is linked to the fact that many migrants, especially the recently arrived, relied on formal and informal support networks and community organizations to help them with language issues, the understanding of health information, and navigating the health system. In general, a better understanding of how the health system works is associated with how well the user can navigate through it [48]. This knowledge of the health system can be derived from either the individuals’ education, socioeconomic level, or even informal support and social networks, and experience throughout the length of stay in the country. It is noteworthy that some migrants face barriers navigating the health system in part related to poor health literacy [49,50,51]. That, adding to the pandemic-related constraints, may have impeded them from appropriate use of the health services.

The limitations of this study must be acknowledged. In the survey, using an interviewer-administered questionnaire to collect data on the perceived impact of the pandemic may have led to biased responses and underestimates of the impact of the pandemic. Furthermore, only three response options were available to assess the perceived impact of the pandemic (“worse”, “same”, and “better” since the crisis) with no further distinction within each response. The findings must be interpreted with caution, bearing in mind that this study used a non-probabilistic sampling approach, therefore the generalizability of our results is limited. Nevertheless, the survey enrolled a large and diverse sample of migrants, including hard-to-reach and socially disadvantaged subgroups such as recent, undocumented migrants, who are frequently understudied. The data was collected during the COVID-19 pandemic providing unique information on the experiences of these vulnerable groups at this exceptional time. The use of quantitative and qualitative approaches allowed a deeper understanding of migrants’ perspectives and experiences, which can help informing policies and strategies to promote a sustainable and equitable health system in Portugal. Future research will be valuable to further understand the role of cultural differences on migrants’ access and use of health services, especially during the pandemic.

## 5. Conclusions

The COVID-19 pandemic intensified inequities in access to healthcare, affecting particularly most vulnerable populations, such as some migrant groups. Our findings indicate a high proportion of migrants not having their health care needs met, mostly those in a more vulnerable socioeconomic situation. Additionally, many remote interactions with health services are likely here to stay. It is crucial to consider additional targeted strategies that provide easy-to-use and accessible resources to facilitate migrants’ access to and navigation of health services, especially those at risk of digital exclusion. This should include involving community-based organizations, community leaders, and informal support networks in the co-design of strategies to ensure adapted solutions that meet migrants’ needs. This study’s findings contribute to a better understanding of the effects of the COVID-19 pandemic on the access and use of health services among some of the most understudied and underserved populations, as migrant groups experiencing vulnerabilities. These findings may be useful to further understand the pandemic adverse impact in similar contexts and migrant populations in other countries, namely in Europe.

## Figures and Tables

**Table 1:** Characteristics of the participants (*n* = 929).

	*n*	%
Sex (*n* = 929)		
Women	506	54.5
Men	423	45.5
Age (*n* = 927)		
18–25 years	141	15.2
26–45 years	616	66.5
>45 years	170	18.3
Education level (*n* = 923)		
Basic education	250	27.1
Secondary education	394	42.7
Higher education	279	30.2
Monthly household income (*n* = 907)		
<650€	591	65.2
≥650€	316	34.8
Length of stay in Portugal (*n* = 927)		
<1 year	171	18.4
1 to 5 years	604	65.2
≥6 years	152	16.4
Migration status (*n* = 915)		
Documented/in regularization	835	91.3
Undocumented	80	8.7
Ability to understand and speak Portuguese (*n* = 925)		
Yes	634	68.5
No	291	31.5
Self-perceived risk to get COVID-19 infection (*n* = 706)		
High risk	145	20.5
Moderate risk	242	34.3
Low/No risk	241	34.1
Do not know	78	11.1

**Table 2:** Characteristics of the participants by perceived change in the access to health services during the COVID-19 pandemic and factors associated with perceived worsening in access (*n* = 929).

	Perceived Change in the Access to Health Services during the COVID-19 Pandemic		Worse Access to Health Services			
	Got Worse *n* (%)	Same as before/Got Better *n* (%)	Crude OR (CI 95%)	*p*-Value	Adjusted OR (CI 95%)	*p*-Value
Total	406 (43.7)	523 (56.3)				
Sex						
Women	252 (49.8)	254 (50.2)	1.73 (1.33–2.26)	>0.001 ***	1.41 (1.02–1.97)	0.040 **
Men	154 (36.4)	269 (63.6)	1			
Age						
18–25 years	63 (44.7)	78 (55.3)	1		1	
26–45 years	259 (42.0)	357 (58.0)	0.90 (0.62–1.30)	0.570	0.77 (0.42–1.34)	0.254
>45 years	84 (49.4)	86 (50.6)	1.21 (0.77–1.90)	0.410	0.75 (0.42–1.34)	0.336
Education level						
Basic education	113 (45.2)	137 (54.8)	1.03 (0.73–1.45)	0.862	0.98 (0.62–1.54)	0.915
Secondary education	169 (42.9)	225 (57.1)	0.94 (0.69–1.28)	0.689	0.82 (0.56–1.22)	0.332
Higher education	124 (44.4)	155 (55.6)	1		1	
Monthly household income						
<650€	281 (47.5)	310 (52.5)	1.58 (1.20–2.10)	0.001 ***	1.43 (1.01–2.03)	0.046 **
≥650€	115 (36.4)	201 (63.6)	1		1	
Length of stay in Portugal						
<1 year	75 (43.9)	96 (56.1)	1.10 (0.71–1.72)	0.662	1.08 (0.61–1.93)	0.784
1 to 5 years	268 (44.4)	336 (55.6)	1.13 (0.79–1.62)	0.516	1.14 (0.72–1.81)	0.591
≥6 years	63 (41.4)	89 (58.6)	1		1	
Migration status						
Documented/in regularization	360 (43.1)	475 (56.9)	1		1	
Undocumented	38 (47.5)	42 (52.5)	1.19 (0.75–1.90)	0.450	1.07 (0.64–1.80)	0.800
Ability to understand and speak Portuguese						
Yes	290 (45.7)	344 (54.3)	1		1	
No	114 (39.2)	177 (60.8)	0.76 (0.56–1.01)	0.062 *	0.70 (0.48–1.01)	0.055 *
Self-perceived risk to get COVID-19 infection						
Low/No risk	90 (37.3)	151 (62.7)	1		1	
Moderate risk	116 (47.9)	126 (52.1)	1.55 (1.08–2.22)	0.019 **	1.64 (1.12–2.41)	0.011 **
High risk	70 (48.3)	75 (51.7)	1.57 (1.03–2.38)	0.035 **	1.59 (1.02–2.46)	0.039 **
Do not know	36 (46.2)	42 (53.8)	1.44 (0.86–2.41)	0.168	1.25 (0.72–2.16)	0.424

CI, confidence interval; OR, crude odd ratio; AOR, adjusted odds ratio. * *p* < 0.1, ** *p* < 0.05, *** *p* < 0.01.

## Data Availability

The data presented in this study are available on request from the corresponding author. The data are not publicly available due to confidentiality reasons.
